# A Comprehensive Systematic Review of the Latest Management Strategies for Hepatorenal Syndrome: A Complicated Syndrome to Tackle

**DOI:** 10.7759/cureus.43073

**Published:** 2023-08-07

**Authors:** Pooja Roy, Naofel Minhaz, Prince Shah-Riar, Sultana Y Simona, Tasniem Tasha, Tahira Binte Hasan, Farhana Karim Abbasi, Farhana Alam, Shamima A Nila, Janifa Akter, Sharmin Akter, Shammo Biswas, Nigar Sultana

**Affiliations:** 1 Internal Medicine, Harlem Hospital Center, New York, USA; 2 Internal Medicine, Dhaka Medical College, Dhaka, BGD; 3 Internal Medicine, Ibn Sina Medical College, Dhaka, BGD; 4 Internal Medicine, Khulna Medical College, Khulna, BGD; 5 Internal Medicine, Rajshahi Medical College, Rajshahi, BGD; 6 Internal Medicine, Armed Forces Medical College, Dhaka, BGD; 7 Internal Medicine, Community Based Medical College, Bangladesh, Mymensingh, BGD; 8 Internal Medicine, Chittagong Medical College, Chittagong, BGD; 9 Internal Medicine, Cumilla Medical College and Hospital, Cumilla, BGD; 10 Internal Medicine, Gonoshasthaya Samaj Vittik Medical College, Dhaka, BGD; 11 Internal Medicine, Bangabandhu Sheikh Mujib Medical University (BSMMU), Dhaka, BGD; 12 Internal Medicine, Shaheed Ziaur Rahman Medical College, Bogura, BGD; 13 Internal Medicine, Sir Salimullah Medical College Mitford Hospital, Dhaka, BGD

**Keywords:** liver transplant, antibiotic in hrs, management of hrs, pathophysiology of hrs, albumin, renla replacement therapy, acute kidney injury, terlipressin, hepatorenal syndrome

## Abstract

Hepatorenal syndrome (HRS), defined by the extreme manifestation of renal impairment in patients with cirrhosis, is characterized by reduced renal blood flow and glomerular filtration rate. It is diagnosed with reduced kidney function confirming the absence of intrinsic kidney disease, such as hematuria or proteinuria. HRS is potentially reversible with liver transplantation or vasoconstrictor drugs. The condition carries a poor prognosis with high mortality rates, particularly in patients with advanced cirrhosis. The latest management for HRS involves a combination of pharmacological and non-pharmacological interventions, aiming to improve renal function and reduce the risk of mortality. Pharmacological treatments include vasoconstrictors, such as terlipressin and midodrine, and albumin infusion, which have been shown to improve renal function and reduce mortality in HRS patients. Non-pharmacological interventions, including invasive procedures such as transjugular intrahepatic portosystemic shunt (TIPS), plasma exchange, liver transplantation, and renal replacement therapy, may also be considered. Though TIPS has been shown to be effective in improving renal function in HRS patients, liver transplantation remains at the top of the consideration for the treatment of end-stage liver disease and HRS. Recent studies have placed importance on early recognition and prompt intervention in HRS patients, as delaying treatment can result in poorer outcomes. Although there are numerous reviews that summarize various aspects of HRS, the recent advancements in the management and pathophysiology of HRS are still insufficient. Therefore, in this review, we summarized a brief pathophysiology and highlighted recent advancements in the management of HRS with a quick review of the latest articles.

## Introduction and background

Over the years, the definition of hepatorenal syndrome (HRS) has changed as its diagnosis is based on many criteria and is mainly considered a diagnosis of exclusion. HRS is defined as a functional decline in renal function in individuals with severe end-stage cirrhosis. It is one of the most severe complications in patients with advanced cirrhosis. According to the 2015 International Club of Ascites (ICA) report, HRS has certain diagnostic criteria [1-9]. According to progression and disease severity, HRS is of two types: HRS-acute kidney injury (HRS-AKI) (previously called type 1), which develops acutely due to liver failure, is rapidly progressive with a bad prognosis, whereas HRS-non-acute kidney injury (HRS-NAKI), mentioned as type 2, is slowly progressive and develops due to refractory ascites. HRS-NAKI is further divided into hepatorenal syndrome-acute kidney disease and chronic kidney disease (HRS-AKD and HRS-CKD). Patients develop oliguria, generalized edema, and circulatory instability with marked systemic vasodilation.

It’s always better to emphasize preventing the risk factors in a hepatically impaired person, as treatment can be difficult and less promising. Risk factors include large-volume paracentesis without albumin supplementation [1], hypovolemia (MAP <80 mmHg [2]) (from any cause like gastrointestinal bleeding, diuretics, lactulose, and poor oral intake) [3], spontaneous bacterial peritonitis (SBP), antibiotic therapy without albumin in treating infection [2], cardiac dysfunction [4], and alcohol overuse [7,8].

After the first year of diagnosis, approximately 20% of patients with advanced cirrhosis will develop HRS, and 40% will do so within the next five years [5]. Children with chronic liver conditions have an HRS incidence of 5% before receiving a liver transplant [7,9]. The estimated annual incidence for HRS‐AKI in the USA ranges from 9000 to more than 35,000. The majority of HRS patients, with a mean age of 64, present in their sixth or seventh decade. The HRS type 1 average age is 62 ± 1 .2 years, while the HRS type 2 average age is 68 ± 1.6 years [2,5,7-9]. Most of the studies found no gender or racial predilection, with a few studies indicating a male preponderance [2,4,5]. The median life expectancy of an HRS1 patient is around two weeks, which is quite devastating [6], and for HRS2, it’s three to six months. Even though the prognosis of HRS is poor, systemic vasoconstrictor (vasopressin, albumin, and newer drugs like terlipressin) or trans-jugular intrahepatic portosystemic shunt is promising. Thankfully liver transplant is the savior here causing a complete reversion of renal function, though liver transplantation is quite challenging for most patients.

Our review highlights the pathophysiology, the updated diagnostic guidelines and management options, including the newest options available for the HRS.

## Review

Research question**

This systematic review aimed to assess how the latest management and treatment guidelines for hepatorenal syndrome compared to previous guidelines in improving patient outcomes. 

Inclusion and exclusion criteria 

**Figure 1:**
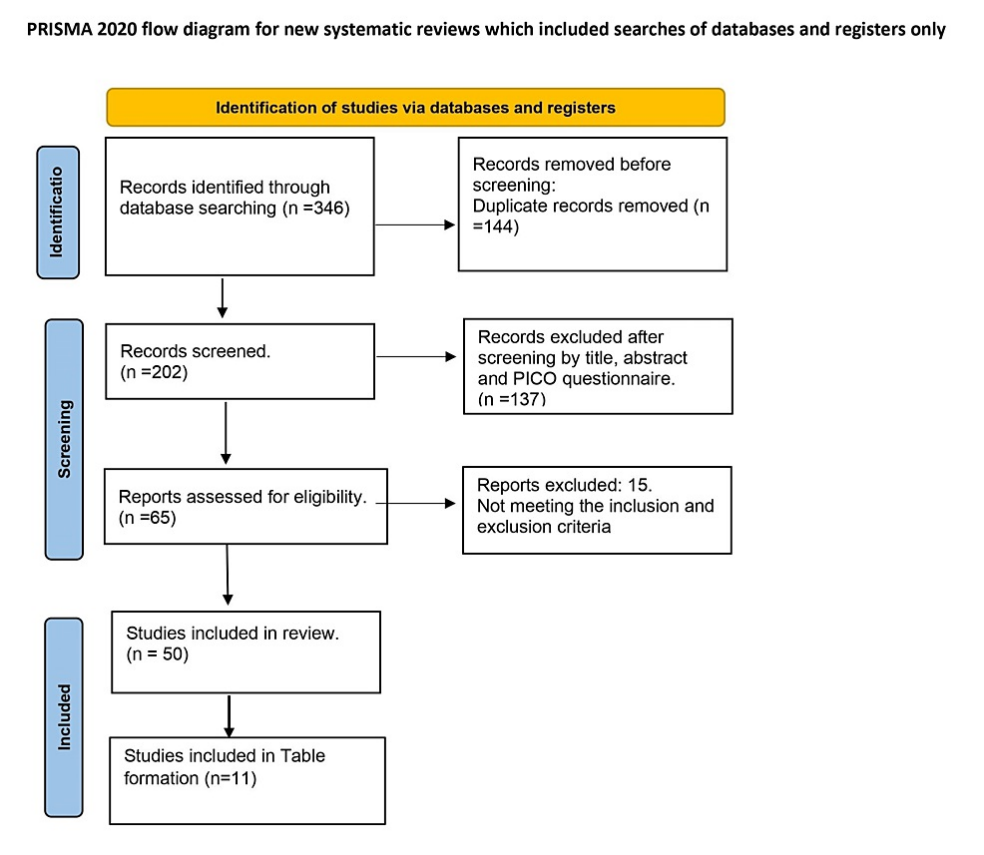
PRISMA flow diagram reflecting the literature review and the total number of articles cited in our review article. PRISMA: preferred reporting items for systematic reviews and meta-analyses.

Related articles were chosen and evaluated using search and MeSH terms. The criteria for including articles in this study were chosen as (1) randomized controlled trials, clinical trials, meta-analyses, systematic reviews, and review articles published/available in English, (2) PICO= (population=diagnosed cases of HRS, intervention=observe the management strategies in the diagnosed cases of HRS, comparison=is terlipressin better than the previously offered management, outcome=follow up on the management outcomes including terlipressin), and (3) published within five years. On the other hand, the criteria for excluding articles were: case reports or case series, editorials, articles published on animal trials, or any ongoing trials (Figure 1).

Our systematic review has some limitations, despite our best efforts. Firstly, PubMed was the only search tool used for this review article. To ensure high quality and prevent data extraction from fraudulent papers and a lack of data extraction tools, we restricted it to PubMed. Our systematic review focuses on the brief pathophysiology to give the reader the justification of the latest addition of terlipressin to the management aid.

Data collection and extraction 

An initial search on PubMed delineated in Figure 1 with ("Hepatorenal Syndrome/complications" (Mesh) OR "Hepatorenal Syndrome/diagnosis" (Mesh) OR "Hepatorenal Syndrome/drug therapy" (Mesh) OR "Hepatorenal Syndrome/prevention and control" (Mesh)), "hepatorenal syndrome" and "hepatorenal syndrome management" found 56, 202, and 56 articles, respectively. After the removal of the duplicates, there were 202 articles to review. A total of 137 articles were excluded after screening by title and abstract, and after applying the inclusion and exclusion criteria, another 15 articles were removed. A total of 50 articles were included in the review article, and 11 articles were included for the table formation (Table 1).

**Table 1:** Articles focusing on the outcome over different management options in HRS. HRS-AKI: hepatorenal syndrome-acute kidney injury, HRS-CKD: hepatorenal syndrome-acute chronic kidney disease, TIPS: transjugular intrahepatic portosystemic shunt, ATN: acute tubular necrosis, eGFR: estimated glomerular filtration rate, SCr: serum creatinine, MELD: model for end-stage liver disease, RRT: renal replacement therapy.

Author name and year of publication	Purpose of study	Type of study	Outcomes and results	Conclusion
Bashir et al., 2019 [10]	To study the management of acute kidney injury in hepatorenal disease	Retrospective cohort study	About 92.3% of patients with HRS received intravenous fluids, and 75.4% received intravenous albumin within 48 h of acute creatinine rise. In this study, the mean age was 55.7 ± 0.61, and baseline serum creatinine was 0.94 ± 0.14, with a diverse racial distribution. About 27.2% of patients met the diagnostic criteria for HRS, and HRS patients had the highest in-hospital mortality rate of 41.5%, compared to 14.1% for pre-renal azotemia and 23.3% for acute renal failure (ARF) (P<0.01).	Early management of AKI in cirrhosis using volume expansion and urinalysis is hindered by delayed HRS-specific therapy, necessitating the development of readily available biomarkers to distinguish AKI causes and standardized treatment to improve outcomes.
Kulkarni et al., 2020 [11]	Safety, efficacy, and pharmacology of terlipressin in patients with cirrhosis specifically	Systematic review		Terlipressin is effective in reversing hepatorenal syndrome (HRS) in cirrhosis and acute-on-chronic liver failure (ACLF) patients, as well as controlling acute variceal bleeding with a mortality benefit. There is a lack of biomarkers to predict terlipressin response; however, continuous infusion through an elastomeric pump or conjugated/depot formulations may offer potential benefits for specific cases of refractory ascites and HRS-2.
Kwong et al., 2021 [12]	To study the effect of norepinephrine in non-ICU settings in the management of HRS	Randomized controlled trial	A total of 61 patients were administered midodrine and octreotide for the treatment of HRS, with a 28% response rate. Among the nonresponders, 20 were administered norepinephrine, of whom 45% achieved a full or partial response.	In non-ICU settings where midodrine and octreotide do not respond, norepinephrine can be used where terlipressin is not available
Ponzo et al., 2021 [13]	To analyze the effect of TIPS in patients with HRS-CKD in the last 12 years	RCT	The study has shown that renal function was significantly improved within one week of TIPS (p<0.001).	This study evaluated the effects of TIPS on renal function in cirrhotic patients with refractory ascites and HRS-CKD. TIPS led to significant and rapid improvement in renal function, indicating a reversible component of HRS-CKD. Although complete normalization of renal function was not achieved, TIPS showed efficacy in improving both renal function and ascites management, independent of baseline eGFR.
Wang et al., 2018 [14]	To assess if terlipressin is safe and efficacious in the treatment of hepatorenal syndrome	Systematic review and meta-analysis	Terlipressin was more effective than placebo in reversing HRS with a lower mortality rate and no significant difference in HRS recurrence or adverse events. Compared to norepinephrine, there was no significant difference in HRS reversal, mortality rate, or recurrence rate, but adverse events were more common in the terlipressin group.	This review concludes that terlipressin shows promising results for treating hepatorenal syndrome (HRS) in terms of efficacy and safety. It appears to improve hepatorenal syndrome reversal rate and renal function when compared to placebo and other vasoconstrictor drugs, except for norepinephrine, which showed similar effectiveness for HRS management.
Buccheri et al., 2022 [15]	For a better understanding of pathogenesis, diagnosis and treatment options for HRS	Review article	Different short-term (albumin use, antibiotics, large volume paracentesis) and long-term definitive treatment options are discussed.	Preventing HRS involves employing specific interventions such as albumin use, antibiotics, and nonselective β-blocker therapy, tailored to the severity of cirrhosis. Although lacking FDA approval, terlipressin is validated as the first-line pharmacotherapy for HRS in the US, while bridging strategies like RRT, liver dialysis, and TIPS are considered before orthotopic liver transplantation (OLT) or simultaneous liver-kidney transplant (SLK) for definitive treatment.
Habas et al., 2022 [16]	For a better understanding of pathogenesis, diagnosis, and treatment options for HRS	Review article	Pathophysiology and different factors related to HRS are discussed along with available treatment. Options include terlipressin, which remains the most effective medical treatment, and liver and kidney transplants, which are the definitive cure.	This review emphasizes that liver and kidney transplantation remain the definitive treatment option for renal impairment in chronic liver disease, especially HRS, despite its challenges such as cost and limited organ availability.
Khemichian et al., 2021 [17]	Update on new definitions, pathophysiology, kidney function assessment, and treatment options on HRS	Review article	Biomarkers may help identify HRS-AKI, which is associated with the worst outcome in patients with liver cirrhosis. Treatment with vasoconstrictors like terlipressin is the most efficacious, and liver transplantation remains the most important step in HRS management.	Early HRS-AKI detection is vital as minor sCr changes may indicate significant GFR decline. Biomarkers help differentiate HRS-AKI from other types, like ATN. Vasoconstrictors, such as terlipressin, are the primary treatment to reverse HRS, while liver transplantation remains a pivotal step for definitive management.
Flamm et al., 2021 [18]	Analyzed existing treatment options for hepatorenal syndrome	Review article	Patients treated with terlipressin showed significant HRS reversal when compared to those treated with a placebo. Further studies are required on terlipressin to assess the population that can benefit from it and minimize its toxicity.	HRS-AKI is a common complication in patients with advanced liver disease. Terlipressin, which is an approved first-line therapy for HRS-AKI in Europe, can be beneficial for many patients with HRS-AKI if it were to be made available in the USA.
Gow et al., 2022 [19]	Assessed the safety and efficacy of continuous terlipressin infusion (CTI) for treating hepatorenal syndrome (HRS), diuretic refractory ascites, and hepatic hydrothorax in patients awaiting a liver transplant in an outpatient setting.	Prospective observational cohort study	The study involved 23 patients with HRS, refractory ascites, or refractory hepatic hydrothorax who received outpatient continuous terlipressin infusion (CTI) for a median duration of 50 days. There were no serious adverse events reported, and CTI resulted in a significant decrease in median serum creatinine and MELD score, as well as a decrease in the frequency of paracentesis/thoracentesis.	Transplant-eligible and otherwise stable patients can be managed with CTI at home for an extended duration under supervision without adverse consequences.
Saif et al., 2018 [20]	This study compared noradrenaline and terlipressin in managing type 1 HRS.	Randomized controlled trial	This study compared noradrenaline/terlipressin treatments for reversing type 1 HRS. Both treatments had similar success rates, with 53% in group A and 57% in group B achieving reversal. No significant differences were found in serum creatinine and urine output.	There is no difference in the outcome of patients with type 1 HRS treated with noradrenaline or terlipressin. Thus, noradrenaline, which is cheaper, can be used instead of terlipressin.

Discussion

Definition and Diagnosis of Hepatorenal Syndrome

In advanced liver cirrhosis, the renal dysfunction that results from the systemic hemodynamic effects of portal hypertension is known as hepatorenal syndrome (HRS). In 1990, the International Club of Ascites (ICA) defined acute renal failure in cirrhosis as an increase in serum creatinine of at least 50% from the baseline value or ≥1.5 mg/dl [1,21]. In 1996, the ICA defined HRS as a kidney function decline in patients with acute decompensated liver disease and portal hypertension characterized by severe circulatory dysfunction; however, they revised the criteria in 2007 and classified HRS into two distinct subtypes: type 1 HRS and type 2 HRS [22-27].

Type 1 HRS is characterized by sudden deterioration of renal function by doubling of serum creatinine to at least 2.5 mg/dl or a 50% reduction in creatinine clearance to below 20 ml/minute within two weeks [28,29]. Type 2 HRS is less severe than type 1 HRS, has a gradual decline in renal function and features prominently as refractory ascites that are resistant to diuretics [1,22,28].

According to studies conducted on the efficacy of terlipressin and albumin for the management of treatment-type-1 HRS, at the initiation of treatment, the greater the serum creatinine (SCr) value, the lesser the likelihood of a positive response to treatment and poor survival. These findings indicate that delaying until SCr has risen above 2.5 mg/dL may decrease the chance that a patient will respond to treatment [24]. Therefore in 2015, the ICA modified the prior criteria and classifications of AKI in patients with cirrhosis, which is consistent with the Kidney Disease Improving Global Outcomes (KIDGO) classification [1].

New Diagnostic Criteria of Hepatorenal Syndrome

HRS classification was updated by ICA in 2015 along with the revised definition of AKI [29]. The diagnosis of AKI is determined before the diagnosis of HRS in this new classification system. The stages of AKI should be determined when AKI is diagnosed since individuals with cirrhosis have a greater mortality rate as AKI progresses through later stages [26] (Table 2).

**Table 2:** New diagnostic criteria of HRS-AKI. Source: [9,15,24,26]. ICA: International Club of Ascites, NSAIDs: non-steroidal anti-inflammatory drugs, RBCs: red blood cells, HRS-AKI: hepatorenal syndrome-acute kidney injury.

Diagnosis includes both cirrhosis and ascites
Acute kidney injury diagnosis meeting criteria according to the International Club of Ascites-Acute Kidney Injury
Removal of diuretic for consecutive two days and plasma volume expansion with albumin 1 g/kg body weight resulting in no response in renal function
There is no shock and no current or recent use of any nephrotoxic drugs, namely, non-steroidal anti-inflammatory drugs, aminoglycosides, iodinated contrasts, etc.
There is no protein in urine specified as >500 mg/day, absence of microhematuria, namely, >50 red blood cells/high power field and there is no pathology in renal ultrasound that is included in macroscopic signs of structural kidney injury

On the basis of serum creatinine concentrations, the ICA divides AKI into three stages. Stage 1 is defined as an increase of serum creatinine ≥0.3 mg/dL or an increase of 1.5 to two times from the baseline value, stage 2 is classified as an increase of serum creatinine less than two to three times from the baseline value, and stage 3 is marked as an increase of serum creatinine less than three times from the baseline or serum creatinine concentration of ≥4.0 mg/dL with an abrupt increase of ≥0.3 mg/dL or renal replacement therapy initiation [21].

According to ICA guidelines, HRS is diagnosed in a patient with cirrhosis and ascites who has stage ≥2 AKI and lack of improvement despite the withdrawal of diuretics or trial of treatment with albumin for plasma volume expansion (1 g/kg per day with a maximum of 100 g/day) for a total of 48 hours, and has no evidence of other etiologies causing kidney injury (i.e., absence of shock, no current or recent use of nephrotoxic drugs, no macroscopic signs of structural kidney injury, such as the presence of proteinuria, microhematuria, or abnormal findings on renal ultrasonography) [21].

The term HRS type 1 was replaced with HRS-AKI. To facilitate early diagnosis and treatment initiation, both the serum creatinine cutoff value and the time interval for renal function decline were eliminated [29]. HRS type 2 was renamed as HRS-NAKI (HRS-NON-AKI) and is based on the estimated glomerular filtration rate (eGFR) rather than the serum creatinine, which is further divided into two subtypes; HRS-acute kidney disease (HRS-AKD) and HRS-chronic kidney disease (HRS-CKD) [1].

Pathophysiology of Hepatorenal Syndrome and Biomarkers

There are several mechanisms that could cause HRS. The most well-known theory causes alterations in splanchnic vasodilation, which causes peripheral vasoconstriction to rise [2,14,27]. Early in the course of the illness, there is modest splanchnic vasodilation, and decreased systemic vascular resistance is counterbalanced by increased cardiac output. Because of the increased production of vasodilator factors in the advanced phase, vasodilation is more prominent and cannot be countered by an increase in cardiac output. Decreased cardiac output appears to come first in severe cirrhosis with ascites, and then hepatorenal syndrome develops [3,17,28-31].

**Figure 2:**
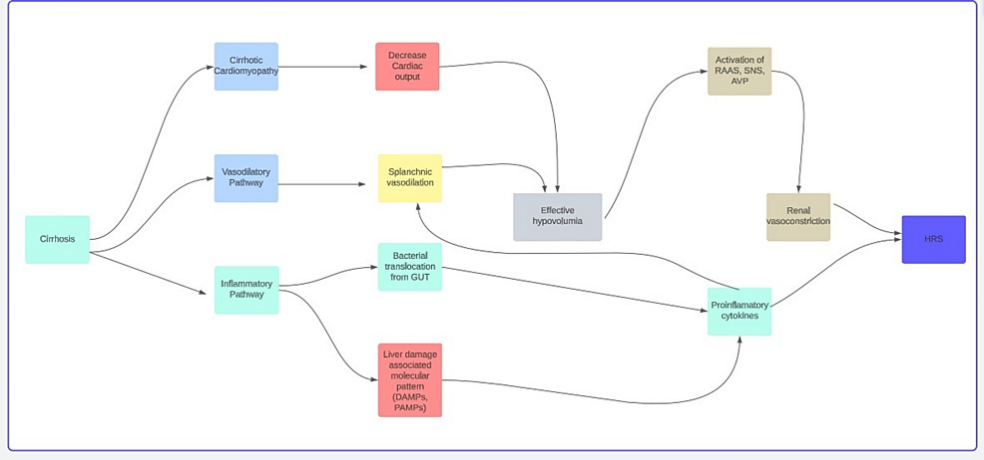
Pathological pathways of HRS development. Source: [21,23-29]. RAAS: renin angiotensinogen aldosterone system, SNS: sympathetic nervous system, AVP: arginine vasopressin, HRS: hepatorenal syndrome. Note: Image is created by own author's creation.

Systemic vasoconstrictor mechanisms are activated (the renin-angiotensin-aldosterone system, sympathetic nervous system, and arginine vasopressin) to maintain arterial pressure, which coupled with increased cardiac output from the hyperdynamic condition helps to sustain renal blood flow (RBF). Even though activating these systems raises arterial pressure, it also causes renal vasoconstriction, sodium retention, which causes edema and ascites, and solute-free water excretion, which leads to hyponatremia and lowers GFR (Figure 2).

The severity of liver disease and portal hypertension are correlated with systemic inflammation in cirrhosis. There is rising evidence that inflammation contributes to hepatorenal syndrome, which has given rise to the idea of a systemic inflammatory illness in cirrhosis. The primary mechanism is the intestinal permeability change that causes the translocation of bacteria and/or pathogen-associated molecular patterns from the gut. Although bacterial infections are frequently the cause of hepatorenal syndrome, about 30% of individuals also have systemic inflammatory response syndrome without having a known bacterial infection [22,29].

Differential Diagnosis Challenges

HRS-AKI is a form of AKI that develops in patients with advanced cirrhosis. However, cirrhotic patients have impaired renal function, which makes them prone to develop AKI due to different causes other than HRS, such as hypovolemia, ATN, or nephrotoxicity. Differentiating these conditions is absolutely needed due to their differences in treatment. Even the prognosis differs as per the etiology of AKI. Hypovolemic AKI shows a better prognosis than HRS-AKI, while structural kidney injury ATN is the worst among these associated with significant mortality [23,27].

Prerenal AKI is renal hypoperfusion without tubular or glomerular injury, which may occur due to GI hemorrhage, dehydration, or diuretic use. It is differentiated from other AKI etiologies by improvement following volume replacement with fluids and/or albumin and withdrawal of diuretics [1,7,9,23]. Patients who do not show response to volume replacement therapy may have other causes of AKI and be evaluated for HRS, structural renal causes like ATN, interstitial nephritis, glomerulonephritis, and post-renal AKI like urinary obstruction [26]. Acute tubular necrosis occurs as a result of an ischemic (e.g., shock) or toxic insult (nephrotoxic drugs). By using the ICA-HRS criteria, intrinsic AKI is differentiated. Prerenal AKI and HRS have urinary Na excretion <20 mEq/L and FeNa <1%; on the other hand, ATN has UNa >40 mEq/L and FeNa >1%. However, there are limitations of this rule due to falsely elevated UNa in cirrhotic patients who are on diuretics. Urinary casts like epithelial and granular cell casts, which are usually seen in ATN, can be present in cirrhosis due to hyperbilirubinemia [21].

Proteinuria and microscopic hematuria should be further evaluated for structural renal causes of AKI with, but not limited to, renal USG, duplex Doppler ultrasonography. Reduced renal blood flow (RBF) can be identified by increased renal resistive indices (RIs) in the renal cortex, medulla, and hilum on duplex Doppler ultrasonography, which may act as an indicator for the development of HRS type 1 and HRS type 2. HRS can progress to ATN due to prolonged vasoconstriction, and it can also occur with prerenal azotemia or CKD. Due to their simultaneous occurrence in cirrhotic patients, differentiation has become a real challenge [26].

Urinary biomarkers, which reflect structural kidney injury, are important for the differential diagnosis of AKI in cirrhosis. Based on several investigated data, the most promising biomarkers are neutrophil gelatinase-associated lipocalin (NGAL), interleukin-8, and albumin.

NGAL is a glycoprotein released by injured renal tubular epithelium that rises significantly in AKI before serum creatinine elevation [24]. A study done on 94 patients with decompensated cirrhosis determined a median uNGAL of 1217.50 in ATN, 465.00 in HRS, and 95.50 in prerenal AKI (P<0.001), and optimal cutoffs of different diagnoses are as defined [21]: acute tubular necrosis (ATN): uNGAL more than 650 ng/mL (100% sensitivity, 83.78% specificity), HRS: uNGAL between 299 and 650 ng/mL (87.9% sensitivity, 96.3% specificity), prerenal AKI: uNGAL less than 299 ng/mL [21].

uNGAL and interleukin-8 are also found to be predictive for prognosis, higher biomarkers are related to higher short-term mortality. During urinary tract infections, uNGAL levels may rise as leukocytes can produce NGAL, so NGAL levels should be interpreted considering these situations [21].

Urinary biomarkers like IL-18, albumin, and liver fatty acid binding protein (L-FABP) were also found to be related with differentiating ATN and HRS. They all showed similar performance indicating the highest levels in ATN compared to HRS-AKI patients or hypovolemia-induced AKI [24].

As demonstrated by different studies, serum cystatin C, which is produced by nucleated cells, maybe a good marker for renal function in cirrhotic patients. A more accurate GFR based on serum cystatin C in comparison to serum creatinine can be drawn, which helps in the accurate and earlier diagnosis of chronic kidney disease [27].

Serum metabolomic profiling, a non-invasive tool, helps to find renal biomarkers, which aid in the early diagnosis of HRS. It can also predict HRS treatment response and renal function improvement after liver transplantation. However, the clinical significance of this metabolomics is still unclear, which warrants further study in this area [26]. In summary, urinary biomarkers showed evidence, particularly NGAL and IL-18, for the differential diagnosis and prognosis of AKI in patients with cirrhosis and warrant further study in this field. Fortunately, there is ongoing research for reliable urinary biomarkers to help in the accurate diagnosis of renal function and HRS-AKI [24,28].

Management

The first step in managing HRS is to treat the cause of hepatic decompensation, which may include managing the primary disease process (i.e., alcohol cessation in severe alcoholic hepatitis or antiviral therapy in hepatitis B infection), using antibiotics judiciously. When immediate improvement in liver function is not possible, medical therapy is the initial step to reverse the acute kidney injury associated with hepatorenal syndrome. Several factors influence the choice of medical therapy, including whether the patient is admitted to the intensive care unit, the availability of specific drugs, national guidelines, and whether the patient is a candidate for liver transplantation [30].

Vasoconstrictor Including Terlipressin (New Addition to the Management) 

Norepinephrine works on alpha-1-adrenergic receptors in vascular smooth muscle cells. Norepinephrine infusion has been shown to increase urine production and improve renal function parameters in patients with HRS. Norepinephrine is frequently linked to cardiac and digital ischemia, which is reversible. Norepinephrine infusion requires intensive hemodynamic monitoring and should only be administered in the intensive care unit (ICU) setting [31]. In the ICU, the first line of treatment for patients with HRS is norepinephrine in combination with albumin. Norepinephrine is administered intravenously as a continuous infusion with the goal of increasing mean arterial pressure by 10 mmHg, and albumin is administered intravenously as a bolus for at least two days.

Vasopressin and vasopressin analogs (ornipressin, terlipressin) bind to V1 receptors on vascular smooth muscle cells, causing vasoconstriction, primarily of the splanchnic and extrarenal circulations, which results in increased effective circulating volume and renal perfusion pressures [18].

Terlipressin is a vasopressin analog that is selective for V1/V2 receptors and has been considered first-line therapy for HRS in Europe (according to the European Association for the Study of the Liver (EASL)) and Asia (according to the Asian Pacific Association for the Study of the Liver (APASL)) [32-34]. Most recently, the FDA approved the use of terlipressin injection to treat adults with hepatorenal syndrome (HRS) with a rapid decline in kidney function [34]. The American Association for the Study of Liver Diseases (AASLD) and the American College of Gastroenterology (ACG) guidelines both recommend terlipressin [33-36].

The CONFIRM trial, a North American-based phase 3 randomized, placebo-controlled trial (n=300) comparing terlipressin to placebo in patients with HRS-AKI-verified reversal of HRS was reported in 63 patients (32%) in the terlipressin group and 17 patients (17%) in the placebo group (P=0.006), supporting that terlipressin, when combined with albumin, is associated with a higher likelihood of reversal of HRS [14,36].

The primary outcome measures to achieve verified HRS reversal, patients had to have two consecutive serum creatinine (SCr) values of ≤1.5 mg/dL, at least two hours apart by day 14 or hospital discharge. With respect to the secondary outcome measures, HRS reversal was reported in 78 patients (39%) in the terlipressin group and 18 (18%) in the placebo group (P<0.001) [37-39]. Abdominal pain, nausea, diarrhea, respiratory failure, and dyspnea are the most common side effects, including raising the possibility of fatal respiratory failure in terlipressin use. Patients with low blood oxygen levels should not begin the medication. During treatment, a pulse oximeter can be used to monitor this. Terlipressin's side effects may prevent patients from receiving a liver transplant. Terlipressin can cause ischemic events, which may necessitate discontinuing treatment. When used during pregnancy, the medication may also cause fetal harm [35].

Midodrine alpha-1 adrenergic agonist is a systemic vasoconstrictor, and octreotide is an inhibitor of the release of glucagon and other vasodilator peptides (which produces splanchnic vasoconstriction); combined therapy theoretically improves renal and systemic hemodynamics [38]. In a retrospective study, 60 patients with hepatorenal syndrome were given midodrine, octreotide, and albumin, while 21 received only albumin. Midodrine and octreotide therapy were associated with significantly lower mortality (43 versus 71 percent) and a considerably higher proportion of patients with hepatorenal syndrome resolution (40 versus 10 percent) [17].

The American Association for the Study of Liver Diseases (AASLD) recommends that vasoconstrictor drugs be continued for up to 14 days after creatinine returns to baseline levels. If the pretreatment creatinine level is high or there is some but not a complete improvement in kidney function after two weeks of therapy, treatment should be extended beyond 14 days to reach the baseline value [34,40-42].

Liver Transplantation 

The curative treatment for HRS is liver or combined liver-kidney transplantation [16,40,43]. The candidates for liver transplantation are selected based on the model for end-stage liver disease (MELD) scoring system [18,41]. This scoring system includes serum creatinine, bilirubin, and international normalized ratio values, which predict 90-day mortality, the mortality rate increases with the score [18]. HRS-AKI is reversible with effective pharmacotherapy and puts them on the waiting list for transplantation in a lower place despite being quite ill, providing a disadvantage to patients with HRS‐AKI on this MELD scoring system [18]. In order to maintain priority for HRS-AKI patients with effective pharmacotherapy, more advances are required [19,40]. The inequality between the donor organ's availability and the recipient waiting for transplantation is also increasing [38,43].

*Renal Replacement Therapy* *(RRT)*

RRT is a temporary treatment option for patients with HRS-AKI awaiting liver transplantation [18,43]. The candidate for RRT is selected based on patient-specific factors [41,44]. Likewise, RRT can be utilized in patients with pharmacologically refractive HRS whose subtype of renal injury is uncertain. On the other hand, for critically ill patients, for example, patients with mechanical ventilation, RRT is inappropriate [40,45]. However, the success rate of RRT in patients with end-stage liver disease presenting with HRS-AKI is unclear [18,41-42]. A recent meta-analysis indicates similar outcomes in patients receiving pre-LT continuous renal replacement therapy and LT recipients without renal failure [41-43]. Another study shows that the longer the patient is on RRT awaiting LT, the more the risk of non-recovery of renal function post-LT [18,44]. Among 1041 LT recipients on RRT during transplantation, the number of prompt recovery of renal function is 707 (67.9%). The shorter duration of RRT in pre-transplant patients leads to a rapid recovery of renal function (15.6 versus 36.6 days; P<0.001). Renal recovery was observed in 70.8% of patients who were on RRT for less than 30 days and 11.5% of patients who were on RRT for more than 90 days [42,46-48]. The best treatment option for HRS with irreversible AKI is combined kidney and liver transplantation.

Antibiotic Therapy

The development of HRS in patients with acute and chronic liver disease can be triggered by bacterial infection [15,18,43]. Bacterial translocation can cause bacterial infection and systemic inflammation, which in turn can cause decompensation in cirrhosis [41]. Preventive treatment with fluoroquinolone and other antibiotics may prevent bacterial translocation and the development of HRS [44,45]. Prophylaxis with antibiotics such as rifaximin and norfloxacin is proven to be effective in the prevention of HRS and spontaneous bacterial peritonitis in high-risk patient populations who have low ascitic fluid total protein concentrations [42,45,46]. However, prophylactic antibiotic use is controversial due to the risk of the emergence of multidrug-resistant organisms. Patients with SBP on prophylactic antibiotics can still develop HRS, which results in mortality or morbidity [47,48]. 

Albumin

Use of albumin in cirrhotic patients may reduce the risk of AKI and mortality and is an important plasma expander in the treatment of HRS-AKI [18,49]. Infusion of albumin increases oncotic pressure, which causes sodium retention that leads to restoration of effective circulating volume and renal perfusion [41]. Intravenous albumin infusion is given at a dose of 1 gm/kg/day as the starting dose for two days, which is followed by infusing 20-40 g daily [50]. Positive renal outcomes along with a decrease in mortality in SBP are seen with short-term albumin therapy [48]. However, there is limited evidence of the administration of albumin in the prevention of HRS in non-SBP infections [47-49]. Intravascular volume overload can occur with excessive infusion of albumin, which can lead to cardiovascular instability, so patients with decompensated liver cirrhosis and portal hypertension need careful monitoring while on albumin [18].

The Molecular Adsorbent Recirculating System (MARS)

The molecular adsorbent recirculating system (MARS) with dialysis is a promising treatment for hepatorenal syndrome (HRS), offering both renal and hepatic support to patients with liver failure. It is an extracorporeal liver support device that removes protein-bound and water-soluble toxins, contributing to the development and progression of HRS [50]. Recent studies have shown that MARS can improve renal function and patient outcomes in cirrhotic patients with acute kidney injury (AKI), including HRS [16,49]. This innovative approach can provide temporary relief for patients who are unsuitable for liver transplantation or waiting for transplantation.

In a retrospective study by Kade et al. (2020), 53 patients with type 1 HRS and alcohol-related acute-on-chronic liver failure (ACLF) were treated with MARS. The study found a 49.1% survival rate at 14 days, with significant improvements in renal and liver function, suggesting MARS as a valuable therapeutic option for such patients [50]. Further research with larger cohorts and control groups is needed to validate the findings and assess the long-term benefits.

## Conclusions

Hepatorenal syndrome is one of the most severe complications in patients with advanced liver cirrhosis. Emphasizing preventing the risk factors in a hepatically impaired person as a treatment option can be difficult and less assuring. Pentoxifylline (PTX) with volume expansion and vasoconstriction is a safe alternative to the standard of care alone. The present review suggested that terlipressin had superiority in improving both hepatorenal syndrome reverse rate and renal function compared to placebo and octreotide in managing HRS. In conclusion, this review provided the best available evidence for the safety and efficacy evaluation of terlipressin for treating HRS with acceptable more drug-related adverse events. The FDA gave approval for terlipressin in HRS-AKI use in September 2022, and the CONFIRM trial supported the approval; however, there is less improvement in terms of mortality and respiratory failure; hence, more trials will strengthen our hypothesis, as reflected in our review.
